# Tertiary Lymphatic Structures in Primary Hepatic Carcinoma: Controversy Cannot Overshadow Hope

**DOI:** 10.3389/fimmu.2022.870458

**Published:** 2022-06-29

**Authors:** Weili Jia, Tianchen Zhang, Qianyun Yao, Jianhui Li, Ye Nie, Xinjun Lei, Zhenzhen Mao, Yanfang Wang, Wen Shi, Wenjie Song

**Affiliations:** ^1^ Xi’an Medical University, Xi’an, China; ^2^ Department of Hepatobiliary Surgery, Xijing Hospital, Fourth Military Medical University, Xi’an, China

**Keywords:** tertiary lymphoid structures (TLS), tumor-infiltrating lymphocytes (TIL), primary hepatic carcinoma (PHC), immunotherapy, cancer prognosis, immune microenvironment (IME)

## Abstract

Tertiary lymphoid structures (TLSs) are organized aggregates of immune cells found in the tumor microenvironment. TLS can influence primary hepatic carcinoma (PHC) occurrence and have an active role in cancer. TLS can promote or inhibit the growth of PHC depending on their location, and although available findings are controversial, they suggest that TLS have a protective role in PHC tissues and a non-protective role in paracancerous tissues. In addition, the cellular composition of TLS can also influence the outcome of PHC. As an immunity marker, TLS can act as a marker of immunotherapy to predict its effect and help to identify patients who will respond well to immunotherapy. Modulation of TLS formation through the use of chemokines/cytokines, immunotherapy, or induction of high endothelial vein to interfere with tumor growth has been studied extensively in PHC and other cancers. In addition, new tools such as genetic interventions, cellular crosstalk, preoperative radiotherapy, and advances in materials science have been shown to influence the prognosis of malignant tumors by modulating TLS production. These can also be used to develop PHC treatment.

## 1 Introduction

Tertiary lymphoid structures (TLSs), also known as tertiary lymphoid organs, ectopic lymphoid structures, induced lymphoid organs, and abnormal lymphoid appendages, are organized aggregates of immune cells such as ectopic central lymphocytes, myeloid cells, and interstitial cells for acquired immune response (1). TLS and secondary lymphoid organs (SLOs) have similar structures and functions (2). TLSs are ectopic lymphatic structures formed from long-term chronic inflammatory stimulation including viral infection, autoimmune diseases, tissue transplantation, and cancer (3). However, the prognostic role of TLS in those diseases is controversial. Previous studies have proven that TLS has a prognostic benefit in metastatic melanoma (4), invasive bladder cancer (5), non-metastatic colorectal cancer (6), endometrial cancer (7), and pancreatic cancer (8). Conversely, there is a reported association between TLS and poor prognosis in lupus nephritis (9), rheumatoid arthritis (10), and other diseases.

Primary hepatic cancer (PHC) includes hepatocellular carcinoma (HCC), intrahepatic cholangiocarcinoma (iCCA), and mixed cancers (HCC + iCCA). HCC is the commonest histological subtype of PHC. As a cancer with an extremely high mortality rate, HCC was the sixth leading cause of cancer-related deaths in 2020 (11). The incidence of PHC is increasing yearly. According to World Health Organization projections, the number of new cases in 2040 will exceed 1.4 million, the number of deaths will exceed 1.3 million, and more than half of the cases will come from Asia (12). Surgical resection and liver transplantation are the main treatment options for early PHC. However, most patients are only diagnosed at the late stages, thus missing the best treatment opportunity (13). PHC is not sensitive to traditional radiotherapy and chemotherapy for malignant tumors. As a result, immunotherapy is one of its promising treatment methods (14). TLS are rich aggregates of immune cells and an important player in tumor immune response. Understanding TLS in PHC may help to further understand PHC immunotherapy. Therefore, this paper reviews recent studies of TLS and briefly introduces the background and concept of TLS. This manuscript focused on the dual impact of TLS distribution in PHC on prognosis, the potential value of TLS as an immune marker, and the potential of TLS formation and other TLS-related factors being treatments for PHC, which can serve as reference information for future research.

## 2 Background of TLS

### 2.1 Definition of TLS

**Figure 1:**
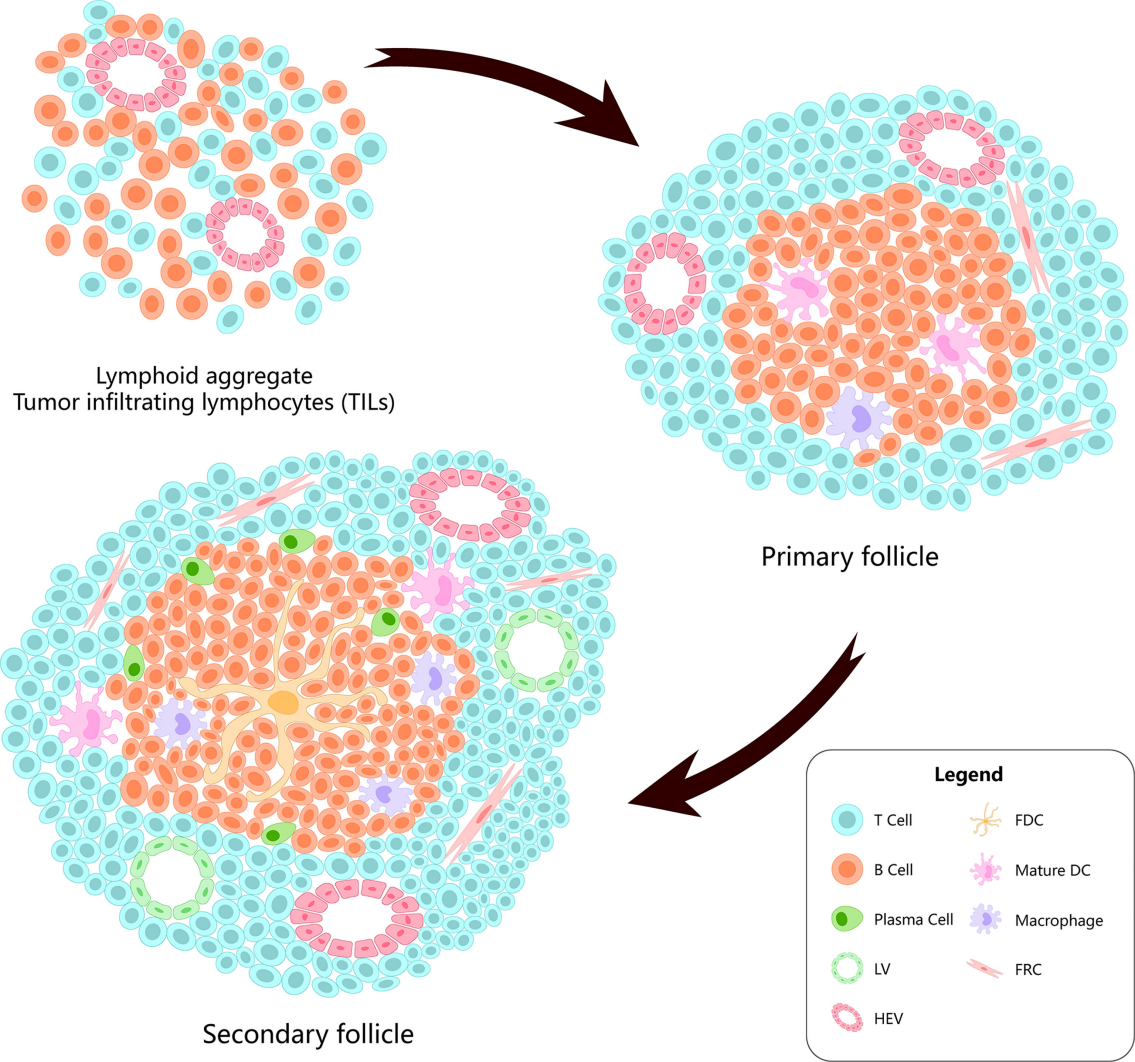
The evolution of the tertiary lymphatic structures.

TLS are lymphocyte aggregates formed in non-lymphoid tissues, which are characterized by B/T cell compartments, differentiated high-endothelial veins (HEVs), and follicular dendritic cell (FDC) networks supporting the germinal center (GC) reaction (15). In TLS, there are T-cell compartments formed by CD3+ T cells surrounding CD20+ B cells. The dominant subsets in the compartments comprise CD4+ Tfh cells (3). Generally, their composition and structure are similar to those of SLO (16) (**Figure 1**).

Tumor-infiltrating lymphocytes (TILs) are produced during the host’s immune response to cancer; however, distinguishing between TILs and TLS remains difficult. Therefore, TILs and TLS are collectively referred to as TLS in this paper. Moreover, there is no approved standard TLS classification. There are several classifications of TLS by different scholars using different criteria (17–19).

### 2.2 TLS and SLO

In the following paragraphs, we compare TLS and SLO. SLO [including the spleen, lymph nodes (LNs), etc.] can proliferate under antigen stimulation and have an immune function—this is an important component of the immune response. They both have similar cell content, matrix composition, lymphoid chemokines, vasculature, and tissue (20). They both have similar functions including the expression of genes encoding lymphocyte chemokines and lymphotoxins (LTs). They usually contain a functional GC, which can mediate *in situ* B-cell differentiation, somatic hypermutation, oligoclonal amplification, and final antibody production (21). Even in mouse models lacking SLO, TLS have been reported to replace the function of SLO completely (22).

Although TLS and SLO have many similarities, they are not the same structures. Morphologically, TLS do not have afferent lymphatic vessels or an envelope like SLO, which means that they can be directly exposed and stimulated by the inflammatory environment. The influence of antigens and cytokines, which may cause FDCs, lymphocytes, and macromolecules to enter TLS without restriction, favor abnormal lymphocyte activation (23). This may be one of the causes of some autoimmune diseases, such as rheumatoid arthritis and lupus nephritis. In addition, TLS and SLO have different formation mechanisms. SLO are formed during embryonic development due to the interaction between lymphoid-tissue-induced cells (LTIs) and stromal cells, while physiological TLS are produced after birth due to the presence of microbiota or the immune response, which has nothing to do with pathology (24).

### 2.3 TLS Formation

TLS formation starts from lymphoid neogenesis (25). In the early stage of formation, innate immune cells (neutrophils, eosinophils, and monocytes) infiltrate the chronic inflammation site rapidly, and monocytes begin to differentiate into resident macrophages. Bone marrow dendritic cells gradually accumulate in leukocyte aggregates, while macrophages are excluded from the developing TLS (3, 23). Simultaneously, B cells upregulate interleukin (IL)-18, chemokine C-X-C motif ligand (CXCL)-13, heat-labile enterotoxin B (LTB), a proliferation-inducing ligand (APRIL), and other lymphoid genes to stimulate T-cell entry (25). From a morphological point of view, TLS formation and maturation can be summarized into three stages. The first stage involves T cells, B cells, and stromal cells, such as FDCs, alpha smooth muscle actin (αSMA), and fibroblasts. The second stage comprises development of polarized clusters of T cells and B cells accompanied by FDCs. The third stage comprises development of mature TLS containing GCs, proliferation of B cells, plasma cells, HEVs, and lymphatic vessels (LVs) (26). Of those, the presence of HEVs is related to the recruitment and activation of naive CD4 T cells (27, 28). LVs help to regulate the immune response around TLS (29).

Several factors regulating SLO formation at the embryonic stage overlap with those regulating TLS formation (30). Stimulation of immune cell infiltration by specific inflammatory factors in inflammatory tissues is also a condition for TLS formation (21). At the initial stage, ectopic expression of TLS-promoting factors secreted by dendritic cells plays a vital role in TLS formation (31, 32). These TLS-promoting factors can jointly recruit and activate LTIs (16). LTIs further produce IL-17, lymphotoxin α-1β-2 (LTα1β2) and bind to lymphotoxin β receptor (LTβR) expressed on lymphoid tissues to form organizers. They further continue to induce the secretion of chemokines, expression of angiogenic growth factor, and expression of adhesion factors IL-17R + stromal cells (33). Cell inflammatory factors and lymphoid chemokines (such as CXCL-13, CCL-21, CCL-19, and CXCL-12) secreted by LTIs can coordinate early recruitment of T cells and B cells and form simple aggregates (34). The secretion of vascular growth factors [such as vascular endothelial growth factor A (VEGFA) and C (VEGFC)] plays a vital role in HEV formation (16). Vascular adhesion factors [such as vascular cell adhesion molecule 1 (VCAM1) and mucosal addressin cell adhesion molecule 1 (MADCAM1)] help cells to remain in TLS for a long time (16). With continuous inflammatory stimulation, the TLSs mature. At this time, T- and B-cell compartmentalization occurs; CD21 + FDC networks appear at the TLS center and gradually form a functional GC (35). Of note, although LTIs are extremely important for SLO development in most areas of the human body, the latest evidence shows that TLS can still be formed in the absence of LTIs (36).

Generally, TLS formation is coordinated mainly by lymphoid chemokines, cytokines, and adhesion molecules (37). It is worth mentioning that TLS do not exist forever. Their existence is closely related to active tissue damage. When there is no continuous inflammatory stimulation, they dissipate after antigen clearance or tissue repair (38). Among TLS-promoting factors, IL-6 can promote TLS development in Th17 cells containing podophyllol. Meanwhile, IL-27 can limit the size and function of TLS by controlling the multiplication of Th17 cells (35, 39). Th17 cells and Th17 cytokines, such as IL-17 and IL-22, also contribute to the occurrence and development of TLS (40–43). Blocking IL-17 or podophyllin (PDPN) can inhibit the production of TLS, which may be caused by the negative regulation of PDPN neutralization in Th17 cell proliferation and differentiation (44). There is also evidence that TLS can also be formed without Th17 cytokines (45). In addition, TLS formation is also affected by B-cell activating factor (BAFF). Stimulation of myofibroblasts with Toll-like receptor agonists and cytokines can lead to the upregulation of BAFF and CXCL-13, thus promoting TLS formation (46). In addition to the above factors, tissue-specific, migrating mesenchymal stem cells, NCR3/NKp30, CD3 lymphocytes, and CD20 lymphocytes have different effects on TLS formation (47, 48).

### 2.4 Functions of TLS

TLS are mainly developed in non-lymphoid tissues to deal with various chronic inflammatory diseases including infection, autoimmune diseases, and cancer (49). However, they are not found in all patients, even those with the same disease (50, 51).

TLS in human solid tumors are essential for the creation of a favorable immune microenvironment to control tumor development. They are involved in the following immune processes, namely, T-cell initiation, B-cell activation, and differentiation into plasma cells, and serve as a factory for antibody secretion (52). In most cases, TLS in solid tumors are closely related to improved tumor prognosis. It can therefore be speculated that they are one of the sites of action for activated lymphocytes that partake in immune responses (53). However, although TLSs are protective in patients with infection or cancer, they are harmful in patients with autoimmune diseases and transplant rejection (54). In some autoimmune diseases, TLS are abnormal structures that produce an immune response against autoantigens. They can promote autoimmune response, stimulate local production of autoreactive T and B cells, maintain autoantibodies in the pathogenic process, and worsen the disease (55).

## 3 Effect of TLS on PHC Prognosis

### 3.1 Influence of TLS Location on PHC Prognosis

**Figure 2:**
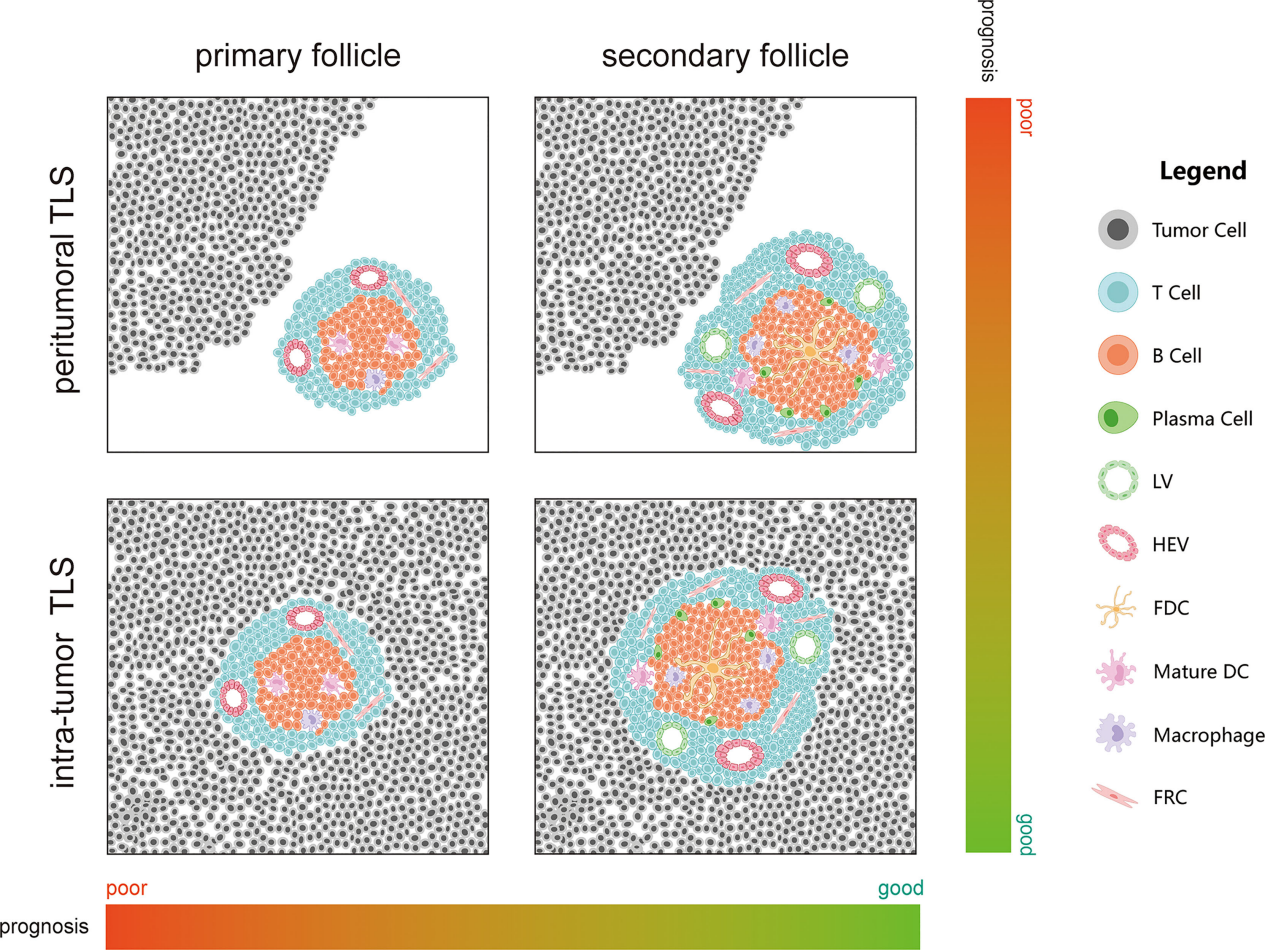
Effect of maturation and location of tertiary lymphatic structures on cancer prognosis.

Many studies have proven that TLS can be used as a cancer prognosis indicator. They are close to or in cancer lesions as immune structures; therefore, they may also play a relatively direct role in anti-tumor immune response (56). From the perspective of liver disease, chronic hepatitis caused by hepatitis B virus (HBV), hepatitis C virus (HCV), alcohol disorders, or other factors is one of the commonest causes of liver cancer, and chronic inflammation is one of the causes of TLS. Therefore, TLSs play a significant role in PHC prognosis (57, 58). In PHC, TLS are found in (intratumor TLS, iTLS) or out of the tumor (extra-tumor or peritumoral TLS, pTLS) and at the junction of the two (**Figure 2**). However, whether iTLS or pTLS is generated eventually is related to the tumor origin or disease stage. Some recent studies have also shown that HCC occurrence is significantly related to genes that promote TLS formation (59, 60). In this study, we summarized the role of TLS in PHC from existing studies (**Table 1**).

**Table 1:** Recent research on TLS in PHC.

Time	First author	Research subjects	Conclusion
2021	Guang-Yu Ding	iCCA/TLS	iTLS contributes to a good prognosis, and pTLS is detrimental to a good prognosis.
2021	Hui Li	HCC/TLS	pTLS contributes to a good prognosis.
2020	Maxime Meylan	HCC/TLS	TLS in early HCC contributes to the development of liver cancer.
2019	Julien Calderaro	HCC/TLS	iTLS contributes to a good prognosis.
2020	Hui Li	HCC/TLS	iTLS contributes to a good prognosis.
2015	Shlomi Finkin	HCC/TLS	pTLS is detrimental to a good prognosis.
2020	Ziying Lin	HCC/TLS	iTLS contributes to a good prognosis.
2021	Fengwei Gao	HCC/TIL	TILs contribute to HCC treatment.
2021	Camila C Simoes	HCC/TIL	TILs contribute to HCC treatment.
2020	Yue Shi	HCC/TIL	TILs contribute to HCC treatment.
2019	Georgi Atanasov	HCC/TIL	TILs contribute to HCC treatment.
2015	Anthony W H Chan	HCC/TIL	TILs contribute to HCC treatment.
2020	Man Liu	HCC/TIL	TILs contribute to HCC treatment.
2019	Hyo Jeong Kang	HCC/TIL	TILs contribute to HCC treatment.
2019	Xuezhong Xu	HCC/TIL	HCC with low TIL has a worse prognosis.
2017	Wei Yao	HCC/TIL	TILs contribute to HCC treatment.
2018	Shigeki Nakagawa	HCC/TIL	HCC with low TIL has a worse prognosis.
2017	Marta Garnelo	HCC/TIL	TILs contribute to HCC treatment.
2015	Shan-Shan Jiang	HCC/TIL	TILs contribute to HCC treatment.
1998	Y Wada	HCC//TIL	TILs contribute to HCC treatment.
1995	K Shirabe	HCC/TIL	TILs contribute to HCC treatment.

#### 3.1.1 Role of iTLS in PHC

In HCC, iTLSs are related to a good prognosis and can reduce the risk of early recurrence of HCC. Moreover, the upregulation of iTLS-related genes can also reduce the risk of HCC recurrence in the early and middle stages, which indicates that iTLS may be related to the existence of sustained and effective anti-tumor immunity (49, 61–64). In addition, the risk of HCC recurrence is also related to TLS maturity (primary or secondary follicles and lymphoid aggregates) (61, 64). More mature TLS (lymphoid follicles) are correlated with prognosis than less mature TLS (lymphoid aggregates) (62). Moreover, TILs above 50% in the tumor parenchyma, also known as “lymphocyte-dominated cancer”, have the best prognosis in HCC (65). As mentioned earlier, the dominant subpopulation in TLS is the CD4+ Tfh cells. In patients with HCC, a decrease in circulating and/or tumor-related Tfh cells is associated with decreased disease progression and disease-free survival (DFS) (66). It has been shown that Tfh cells can drive resident CXCR5 effector T cells to produce Th1-directed responses in active TLS (67). Th1 is the most detected Th cell subset in non-cancerous tissues, and Th2, which is widely detected in patients with a poor prognosis, is considered to have a cancer-promoting effect (68–70). Th1 cells may be related to the initiation and/or maintenance of local and systemic adaptive immune responses while protecting patients from tumor invasion and metastasis (71). Some scholars believe that the response rate to immunotherapy will improve if harmful Th2 cells are modified to protective Th1 cells (72).

Studies on TILs and non-tumor hepatic lymphocyte infiltration (LILs) of HCC have shown similar results. Patients with more TILs have a better prognosis than those with fewer TILs. Contrarily, patients with more LILs in adjacent tissues have a worse prognosis than those with fewer LILs (73–75).

#### 3.1.2 Role of pTLS in PHC

Unlike iTLS, pTLS can promote HCC occurrence (76, 77). In HCC, the TLS density of non-neoplastic liver tissue around HCC seems to be related to poor prognosis, especially late recurrence and death after HCC resection (62, 77). pTLS are also related to *de novo* HCC in patients with chronic inflammation and fibrosis or cirrhosis, and with the increase in TLS, the prevalence of HCC in such patients will also increase (62, 77). Finally, it was also found in mouse models that depletion of TLS in non-neoplastic liver parenchyma is helpful to inhibit HCC progression (77).

TLS can also be detected in early liver lesions (EHLs) such as high-grade atypical hyperplastic nodules, early liver cancer, and advanced small liver cancer (58). As the tumor progresses, these early lesions gradually transform into tumor tissue and can be regarded as paracancerous tissue. EHLs show TLS formation, immune activation, and overexpression of carcinogenic factors; however, this immune response cannot prevent cancer progression. Furthermore, the expression of such immune checkpoints and immunosuppressive molecules may lead to immune escape (58). The overall density and PD-L1 expression of immune cells in EHL are higher than those in their corresponding surrounding tissues—the average density of CD4+ T cells and CD8+ B cells increase three times (58). A high PD-L1 expression leads to immune escape of tumors and, hence, cancer progression (78, 79). The number of TLS increases with the age of the patient, and the accumulation of cancer progenitor cells in TLS is detected. These progenitor cells, which are presumably tumor-inducing cells, can imply a strong tumor-promoting effect of TLS (80). In addition, studies have shown that lymphoid neogenesis depends on LTβR signaling, and TLS can provide paracrine LTb signals to early progenitor cells to promote their growth (71). The progenitor cells expressing E-cadherin in TLS can promote HCC occurrence. After these cells are inhibited, the TLSs are suppressed, and the incidence of HCC is also reduced, which also proves that progenitor cells depend on the adaptive immune system (71). No morphologically mature TLS has been found in EHLs; most TLS in EHL lack CD21 FDCs, and their anti-tumor ability is not perfect. The occurrence of precancerous lesions can be limited by the anti-tumor ability of TLS in EHLs (58).

Coincidentally, in iCCA, the survival time of patients with TLS in the tumor’s surrounding is poor, while the TLS in the intratumor area is positively correlated with a good prognosis (49). Regulatory T cells (Tregs) in TLS can inhibit endogenous anti-tumor immune response; therefore, an increase in the number of Tregs is closely related to the low survival rate of patients. This can lead to worsening of the disease and an increase in tumor infiltration by CD4+ and CD8+ T cells and macrophages (71). TLS-related Tregs can also help tumor immune escape by inhibiting the endogenous immune response against the tumor simultaneously (57, 81, 82). In addition, compared with pTLS, although Tregs in iTLS are significantly increased, Tregs are only related to poor prognosis caused by pTLS (49). Finally, while patients with immune activity have the lowest prognostic risk, patients with immune resistance have the highest prognostic risk (49).

The findings in a study by Finkin et al. suggest that the poor prognosis is caused by pTLS (77). They used transgenic mice to explore HCC development. First, mild inflammatory response and TLS formation were observed in mice, and then, HCC appeared in these mice. The researchers first identified HCC progenitor cells with double-positive GFP and E-cadherin in TLS. Next, these progenitor cells migrated out of the cell and developed HCC outside the cell. Therefore, pTLS may play a similar role with tumor factories in HCC, leading to a poor prognosis of patients with HCC, which is not surprising.

However, the role of pTLS remains controversial. In a study by Li et al., pTLS density was related to good prognosis, that is, there was a positive correlation between high pTLS density and better overall survival (OS) and recurrence-free survival (RFS), and the survival rate of patients with a GC was higher than that of patients without a GC (81). They also observed that pTLS was significantly related to an increase in CD3+ T cells, CD8+ T cells, and CD20+ B cells in tumors, and also to a decrease in infiltration of Foxp3+, Tregs, and CD68+ macrophages (81). This is inconsistent with the increase in Tregs caused by pTLS described earlier, and a clear association has been found between Tregs and the pathogenesis of HCC and even other cancers (57, 81, 82). Tregs play a strong immunosuppressive role and promote immune escape (83, 84). The decrease in Tregs in the results suggest pTLS weakening on suppression of the tumor microenvironment (TME), which may be one of the reasons for the discrepancy between the findings by Li et al. and those of others. Regarding the specific reason for the decrease in Tregs in the study by Li et al., further research is needed. This may explain the controversial findings. In addition, previous studies have proven that CD20+ B cells can promote Tregs proliferation. Conversely, Li et al. reported that high CD20+ infiltration is accompanied by a decrease in Tregs infiltration (85–87). In fact, the role of CD20+ cells remains controversial (88). So far, it is speculated that CD20+ B cells may have a dual effect on HCC; this can be a useful point of focus for HCC treatment research.

### 3.2 Effect of TLS Cellular Components on PHC Prognosis

The function of TLS depends on their structure—rich in immune cells and in direct contact with cancerous tissue. Therefore, it is speculated that the cellular components of TLS have a relatively direct impact on the prognosis of PHC, and our findings seem to confirm this. In TLS, patients with high CD3, CD8, and NK cell infiltration have a better prognosis (83). High levels of CD3 and CD8 infiltration in TILs have a better prognostic value for OS. Higher levels of CD3, CD4, and CD8 imply better DFS and recurrence-free survival (RFS), while high levels of FoxP3 represent worse OS and DFS/RFS. High CD4 percentage and high CD4/CD8 ratio also affect the OS of patients. In addition, FoxP3/CD4 and FoxP3/CD8 ratios are negatively correlated with OS and DFS/RFS (89–92). Further studies have shown that there is a correlation between the density of B cells in TILs and the density of T cells, and the survival rate of patients with HCC having higher densities is higher. The co-expression of CD27 and CD40 on T cells and B cells is related to the survival rate of patients, and the density of B cells is related to the activation of T cells and NK cells and anti-tumor effects. In addition, the relationship between CD20+ mature B cells and tumor suppression is still controversial (88). However, most of the evidence so far supports that CD20+ B cells weaken tumor suppression (85–87). Studies have shown that CD39 may be involved in regulating the inhibitory ability of tumor-invasive CD8 + Tregs (93). However, high-affinity new antigens (HANs) are positively correlated with higher frequencies of CD39 and CD8 TILs, and patients with a higher HAN value have a higher anti-tumor activity (94). The activation of CD40 cells can significantly enhance the response to anti-PD-1 treatment (95). Regarding macrophages, the density of CD38 and CD68 macrophages is related to the improvement of postoperative prognosis, while the total density of CD68 macrophages is related to poor prognosis (96). High expression of reverse transcription cysteine-rich protein (RECK) was associated with more TILs and a significantly better prognosis than in patients with low RECK expression (97). The expression of T-cell activation (VISTA) protein in HCC showed cell specificity, and its expression is significantly correlated with CD8+ TILs. Patients with both VISTA+/CD8+ cells have a more favorable TME and better OS (98). FOXP3 expression in natural T cells is helpful in obtaining effective immunosuppressive ability, and FOXP3 Tregs/CD4 T cell ratio is an independent prognostic factor for OS (99, 100). Tregs promote immune escape in tumors, which inhibits the activity of T cells, bone marrow cells, and stromal cells, leading to T-cell dysfunction (83, 84). WNT/β-catenin inhibitor ICG-001 plus radiotherapy (RT) can promote CD8+ T-cell infiltration and IFN-γ production in TILs and simultaneously reduce the number of Tregs, thus contributing to HCC treatment (101, 102). Joint blockade of TIGIT and PD1 can improve the function of CD8+ TILs that do not respond to single PD1 blockade, thus inhibiting HCC development (103). A simultaneous high expression of PD-L1 and CD8+ TILs is an important prognostic factor related to immune checkpoint pathway in HCC (104). Therefore, these cellular components can be used as predictors of therapeutic effect and deserve further study.

## 4 The Value of TLS for PHC Treatment

### 4.1 The Potential Value of TLS as a Marker for Immunotherapy

Immunotherapy continues to evolve and has become one of the treatments for patients with PHC, but not all patients respond to these treatments. Therefore, the identification of appropriate biomarkers is an urgent need for the treatment of PHC. Recent reports suggest that TLS may be an effective biomarker for immunotherapy.

In renal clear cell carcinoma and melanoma, the presence and density of TLS correlate with the responsiveness of immune checkpoint (ICP) therapy (53, 105). In breast cancer, the expression level of immune checkpoint molecules is related to the level of TILs and TLS formation (106). In addition, a strong correlation between TLS and good ICP treatment outcomes has also been observed in soft tissue sarcomas and bladder cancer (107, 108). Interestingly, immature TLS follicles are observed in most unresponsive patients; therefore, there may be a strong relationship between TLS maturity and treatment. Furthermore, in the study of sarcomas, TLS had a better prognosis in samples with high immune infiltration levels (107). Therefore, it is reasonable to assume that the stage of TLS [no follicles, primary follicles, and mature (secondary) follicles] has a direct impact on the reactivity of ICP. Regarding targeted drugs, there is also evidence to prove the biomarker value of TLS. Treatment of HER2/neu tumors with trastuzumab is associated with better DFS in TLS-enriched tumors (109). In a recent study, the appearance of TLS in immune-checkpoint-resistant PTEN-null prostate cancer is also associated with better treatment outcomes against PI3K inhibitors (110). In gastrointestinal stromal tumors (GIST), high-density TLS is associated with lower imatinib resistance, recurrence, and more favorable survival (111). Patients with drug-resistant GIST have more Tregs, which are one of the inhibitors of the immune system.

In a study on PHC immunotherapy, Vella et al. found that patients with HCC having TLS responded better to ICP therapy with carbosantinib and nivolumab (53). In another HCC study, the authors found that aspartate β-hydroxylase is an ideal tumor-associated antigen and can be used as a target for HCC immunotherapy, but its function is partly dependent on the presence of TLS (112). In addition, patients with a higher expression of TLS characteristic genes such as CCL-5, CXCL-9, CXCL-10, and CXCL-13 will also have a better response to immune checkpoint inhibitors (ICIs) (113, 114). This suggests that TLS can transform the immune microenvironment of patients to some extent, or at least there is a close relationship between the two. The former can affect the cell composition of the latter and provide a suitable environment for the T-cell-mediated ICP response and ultimately affect the effect of immunotherapy.

### 4.2 The Value of Modulation of TLS Formation for PHC Therapy

Tumor-associated TLSs are usually associated with a good prognosis for most cancer types; tumor-associated TLS and chronic intratumoral inflammation are associated with tumor immune tolerance, suggesting that TLS may increase cancer invasiveness (115). A successful anti-tumor immune response cannot be achieved without the synergistic effect of the body’s immune cell components. TLS formation may be related to the relationship between immunosuppression and recovery of immune cell function. The recovery of cell function activates anti-cancer immune-related pathways and then induces TLS formation. After TLS formation, it can further strengthen the anti-tumor effect and improve patient survival. Many attempts to modify TLS formation as a treatment for cancer have been made; these include chemokines and immunotherapy. These approaches are further reviewed in this section.

#### 4.2.1 TLS Regulation by Chemokines/Cytokines

Modern information technology, bioinformatics, has become a powerful tool for identifying biological phenotypes. Some recent studies have used these tools to screen TLS-related chemokine genes, which provide favorable conditions for analyzing the role of chemokines in TLS formation. The unique 12 TLS chemokines (CCL-2, CCL-3, CCL-4, CCL-5, CCL-8, CCL-18, CCL-19, CCL-21, CXCL-9, CXCL-10, CXCL-11, and CXCL-13) can affect TLS status directly and further affect patient prognosis. For example, patients with a low chemokine expression in colorectal cancer have a poor prognosis (116).

B-Lymphocyte chemokines can lead to the formation of LN-like structures, such as HEVs, interstitial cells, B cells, and T cell compartments (117). These LN structures can directly induce TLS formation, thus assisting the treatment of cancer. Different chemokines have different abilities to induce immune infiltration. For example, the CCL21 induction ability is stronger than that of CCL19 (118). CCL21, as a T-lymphocyte inducer, has been reported to recruit T cells into TLS through CCR7 to promote the formation of TLS. Some studies have found that the combined application of IL7 and CCL21 can improve the anti-tumor efficacy of various solid tumors (119). In addition, CCL21 combined with anti-CD25 monoclonal antibody can improve anti-tumor efficacy in HCC. Therefore, CCL21 may be one of the feasible targets for TLS induction and anti-PHC.

Hox antisense intergenic RNA (HOTAIR) is related to poor HCC prognosis. It can promote CCL-2 secretion and may participate in the recruitment of macrophages and bone-marrow-derived suppressor cells to the TME (120). The famous Chinese medicine Gehua Jiecheng Decoction can inhibit the expression of CCL-2 in the liver while effectively inhibiting the development of tumor cells and reducing the tumor area. In addition, the expression of inflammatory factors and angiogenesis factors in the tumor is also reduced to varying degrees to antagonize the immunosuppressive effect of the liver cancer microenvironment (121). There is also evidence that blocking CCL-2 can promote TME recovery from inhibition (122). The serum CCL-3, CCL-4, and CCL-5 levels of patients with HCC are increased and closely related to activated circulating monocytes (123, 124). The serum CCL-3 levels of patients with HCC and a good response to regorafenib chemotherapy are reduced (125). Studies have shown that CCL-5 can activate and recruit M2 macrophages in HCC, increase the proportion of M2/M1 macrophages, and promote HCC progression (126, 127). However, in a study on Biejiajian Pill and yttrium-90 (Y90) radioactive embolism (RE), CCL-5 expression was found to significantly suppress tumor cells (128, 129).

LTβR is a cell surface receptor, which is involved in apoptosis and cytokine release. Studies have shown its key role in LN formation. LTβ deficiency leads to serious defects in the development of lymphatic organs in mice (130). Regarding the similarity between TLS and SLO, TLS can be induced or inhibited by LTβR. DCs, as a source of LT, are closely related to chemokines that contribute to TLS formation. DCs can also promote LT signaling through LTβR to achieve HEV differentiation and LN function (131). DCs promote the infiltration of immature T cells and NK cells into the TME and prolong the OS of mice when injected into their abdominal cavity (132). This proves that to induce TLS formation, the expression of chemokines can be increased through DC. LIGHT/TNFS14 is an LTβR ligand, which is expressed on immature DCs and activated T lymphocytes. By inserting the gene encoding LIGHT into attenuated *Salmonella typhimurium*, its oncolytic activity is strengthened, thus inhibiting the growth of the primary tumor and lung metastasis dissemination (133). Perhaps, attenuated or non-toxic bacteria have similar therapeutic effects with DC injections, and the advances in genetic engineering can also cause a change in required therapeutic proteins to form an artificial antibody factory in the body. In HCC, LTβR signaling is involved in the occurrence of HBV-related hepatitis and HCC (134, 135). Meanwhile, the LTβR pathway inhibits TLS formation (136). This may be achieved by regulating the production of pTLS in the liver. In addition, some studies have shown that overexpression of BCL-2 in HCC cell lines can enhance LIGHT-mediated apoptosis in Hep3BT2 cells and thus inhibit tumor cells (137). Studying the effect of LTβR in PHC tumors may bring new discoveries to the role of LTβR in PHC.

Interestingly, promoting or inhibiting TLS formation in HCC plays a favorable role in prognosis; the reason for this difference is unclear. However, we speculate that it may be related to the distribution of its own TLS inside and outside the tumor—whether the increase in TLS markers that do not exist in cancer lesions, such as chemokine CCLs in serum, can be regarded as an increase in TLS in adjacent tissues, which corresponds to the role of pTLS mentioned above. On the other hand, there is no doubt that the *in vivo* immune response requires the coordinated development of multiple factors including various complex signaling pathways, or cell-to-cell crosstalk, and these experiments on immunosuppressed mice did not obviously consider this situation. Using humanized immune mice to transplant patient-derived tumor tissue for related research may be one solution. Finally, as described above, abnormalities in the composition and function of cells in TLS can affect the disease course, which is not reported in recent studies. However, there is a recent study that verified these conjectures. The author found that upregulation of CXCL13 can promote immune escape in HCC, but under the combination of vaccine and PD-1 inhibitor, CXCL13 produced by cancer cells can recruit T lymphocytes into TLS and have a positive anti-tumor effect (112). Obviously, CXCL13 has contrary roles before and after processing. This may be explained by the aforementioned reasons. In conclusion, these results suggest that chemokines involved in TLS formation can be used in combination with various therapeutic methods. Further research on their use in induction of TLS formation or inhibitory targets to improve HCC prognosis is required.

#### 4.2.2 TLS Regulation by Immunotherapy

There are several advances in immunotherapy in recent years. Some of them can promote TLS formation while producing therapeutic effects, which may confer dual anti-tumor effects on immunotherapy. ICIs can induce TLS formation and play an important role in the formation of TME with anti-tumor properties. In a study on the treatment of non-small cell lung cancer (NSCLC) with an ICI (nivolumab), TLS formation was observed in the samples of cases that responded to ICIs, unlike in the samples of unresponsive cases (138). Furthermore, TIL infiltration was more in biopsy samples from patients with advanced melanoma receiving anti-PD-L1 therapy (139). In addition, in another study using the allogeneic PDAC vaccine (GVAX) combined with low-dose cyclophosphamide to reduce Tregs as a treatment, upregulation of TLS infiltration after GVAX was observed (53).

As mentioned earlier, iTLS can inhibit the progression of PHC. The transcription factor CEBPA is the main regulator of liver homeostasis and bone marrow cell differentiation. Some studies have found that upregulation of CEBPA gene expression can induce the formation of iTLS in the TME and tumor suppression (140). Neoadjuvant drugs, cabozotinib and nivolumab, can also promote iTLS formation and inhibit HCC occurrence (53). In addition, ICIs are promising drugs for the treatment of advanced HCC. After HCC is treated with anti-PD-1 antibodies, TILs and patient survival (PFS and OS) increase (141). GVAX has been proven to induce iTLS formation (53). Selective internal radiotherapy can also significantly promote the recruitment/activation of effector immune cells in tumors and TIL formation (142). This shows that ICIs can convert non-immunogenic tumors into immunogenic tumors through TLS; it also suggests that the combination of ICIs and vaccine-based immunotherapy can be a potential treatment strategy.

In addition, inhibitory TME has an important influence on tumor immunity. Some scholars have found that in non-HBV/non-HCV HCC, although TILs secrete IFN-γ, they cannot kill cancer cells (143). Therefore, the conversion of inhibitory TME into immunoactive TME is a key focus for HCC treatment. Some studies have found that IL-2 can restore the anti-tumor activity of TILs (144). Activation of IL-12-mediated pathways often represent a better prognosis (145). It should be studied as a dynamic marker of the functional state of CD8 TILs. Another study also showed that the expression of TIM-3 and/or PD-1 on TILs will impair their function. They also reported that blocking TIM-3 or/and PD-1 can reverse the dysfunction of TILs in HBV-related HCC (146).

After years of research, tumor vaccine is now one of the feasible tools for induction of TLS formation. In addition to the GVAX mentioned earlier, in another study involving patients with pancreatic cancer receiving granulocyte-macrophage colony-stimulating factor (GM-CSF) vaccine, TLS was shown to regulate adaptive immunity (53). The production of TLS, which is highly related to CD8+ T cells and Th1 infiltration, can also be observed in patients with cervical cancer treated with HPV vaccine (147). These changes are characterized by increased expression of genes related to immune activation and effector function. The study of another vaccine, Nano-sapper, reported its ability to suppress the inhibitory effect of TME in pancreatic ductal adenocarcinoma mouse tumors and induce intratumoral TLS production to improve prognosis (148). Therefore, removing inhibitory TME is key in inducing TLS and improving prognosis.

#### 4.2.3 TLS Regulation by HEV Induction

HEV is a specialized posterior capillary vein with structural and functional differences from normal blood vessels. It is found in SLO and TLS. It affects TLS formation by mediating lymphocyte migration (53). HEV in human tumors is related mainly to increased survival rate. Tumor HEV (TU-HEV) in mice has been shown to cultivate lymphocyte-rich immune centers and enhance immune response when combined with different immunotherapy drugs, which is often considered as a key factor in TLS formation. In a retrospective study of primary breast cancer, TU-HEV density was positively correlated with DFS, metastasis-free survival, and OS (149). Therefore, it can be proven that it is of great significance for anti-tumor immune activity. Moreover, some scholars observed MECA-79-positive blood vessels in melanoma, breast cancer, ovarian cancer, colon cancer, and lung cancer samples (53). These findings were subsequently verified by other scientists and successively confirmed in kidney, stomach, pancreas, and head and neck cancers (53). In many diseases, the intensity of MECA-79 is correlated with the degree of monocyte infiltration in the lesion, and the density of MECA-79 + HEV cells is also positively correlated with clinical parameters. In primary melanoma, MECA-79 + HEV cells are associated with reduced tumor invasiveness (53). The combination of high-density MECA-79 + HEV cells and CD8+ T cells is also a prognostic factor for OS in gastric cancer (150). In addition, MECA-79 is more expressed in the normal bile duct epithelium in iCCA, which implies that MECA-79 expression is inhibited in tumor tissues and may further affect the production of HEVs and iTLS (151). Therefore, MECA-79 may be an implicit powerful target for PHC therapy.

### 4.3 Other Factors Related to TLS That May Affect Treatment

There are fewer studies on the generation and function of tumor TLS than on TLS therapeutic value. The tumor mutation burden (TMB) represents the number of DNA mutations per million bases (Mut/Mb) sequenced in a particular cancer. As the number of mutations and new expressions increases, these new antigens may be immunogenic and trigger T-cell responses. Initially, TMB was identified as a biomarker for ICIs, while few recent studies point it to TLS. In a study using computers to predict new antigens for pancreatic ductal adenocarcinoma, it was pointed out that the TMB of early TLS tumors is low. However, mature TLS with GCs have significantly more restrictive new antigens expressed in samples with a better prognosis (53). Some scholars have used public databases and found that tumors with a high TMB have a higher TLS density in NSCLC and melanoma based on 12 chemokine characteristics (152). Both diseases have good responsiveness to ICIs. These studies show the importance of other molecular features apart from pure TLS maturity stage and density for cancer treatment. In fact, we speculate that highly unstable tumor tissues with a high mutation probability are more likely to trigger the expression of new antigens.

Mutations in specific genes also play an important role in the occurrence and development of TLS. BRCA-mutated tumors have a strong infiltration of CD8+ T cells, and their mutations are positively correlated with high TLS scores in many tumors (including breast cancer, prostate cancer, or endometrial cancer) (53). On the other hand, some other mutations such as CTNNB1 and IDH1 are negatively correlated with high TLS scores. These mutations may directly or indirectly participate in TME formation and eventually affect TLS formation.

Various immune components in TME are mixed, and there may be some crosstalk between various effector cells (CD8+ T cells or NK cells) involved in eliminating tumors or between them and tumor cells, thus affecting their effect of fighting cancer. There is evidence supporting this. One study of HCC showed that tumor cells are able to release YWHAZ (aka 14-3-3ζ) in the TME *via* crosstalk to inhibit the anti-tumor function of tumor-infiltrating T cells (153). Blocking YWHAZ may promote HCC treatment.

Second, some researchers found that there is a difference in the transcriptional characteristics of T cells extracted from tumors with and without TLS and from the tumor TLS itself (53). Third, B cells can function as antigen-presenting cells to promote T-cellular immunity (154). In addition, the study also found that T cells in tumors seem to be a prerequisite for B-cell infiltration (53), which supports the fact that T cells are involved in the recruitment of B cells and TLS formation.

The administration of preoperative radiotherapy and chemotherapy (neoadjuvant chemotherapy, NAC) is related to the increase in TLS formation. In hepatoblastoma with a better prognosis than adenomatous *Escherichia coli* mutation, a significant increase in TLS formation can be seen in tissues before and after cisplatin chemotherapy (155). Patients with pancreatic ductal adenocarcinoma have a better prognosis after NAC, while TLS increase (156). However, during NAC, the formation of TLS is generally impaired, which is manifested by cell reduction and area reduction, but 2 weeks after treatment, their function and size normalize gradually (157). This phenomenon may be related to tumor cell death caused by radiotherapy and chemotherapy. After tumor cells die, they release the new antigens they carry, and the DC captures these antigens and triggers a stronger anti-tumor immunity.

The introduction of materials science has made biomaterials an alternative for disease treatment. Biomaterials are expected to modulate TLS formation through controlled chemokine release. Hydrogel can deliver antigen chemokines and cytokines to DCs to induce cell response (158). Research on artificial LNs has been ongoing for many years (159). Regarding future tumor therapy, the development of biomaterials will be a powerful assistant.

STING (STimulator of INterferon Genes) is a cytoplasmic DNA-sensing protein, which has strong pro-inflammatory ability in tumor-associated stromal cells and can upregulate the expression of various cytokines, which can also be a way to induce TLS formation. In melanoma, STING activation in the tumor can promote the normalization of tumor vessels, enhance lymphocyte infiltration, and promote the formation of local TLS (160). In mouse models of breast cancer, lung cancer, and melanoma, low-dose STING agonists can coordinate the promotion of tumor vascular normalization and CD8+ T cells to control tumor growth (53). The combination of STING agonists with other treatments may be a candidate for improving TLS-related anti-tumor immune response.

## 5 Discussion

Research on TLS in PHC is still at the initial stage; there are few related studies. Regarding PHC, while the positive effect of iTLS is sure, pTLS might have a negative effect. There are several reports on the positive role of iTLS in various cancers; therefore, we can speculate that they are important in PHC. However, there are no large-scale multicenter studies on siTLS, creating the need for further research. However, there are several limitations that need to be overcome for research on TLS in PHC and even in all cancers.

First, the studies used different TLS evaluation criteria. Furthermore, the TLS determination methods were different, time consuming, subjective, and difficult to use in clinical practice. The lack of reproducible and standardized TLS identification methods is a major setback. However, the advances in artificial intelligence and computers have made it possible to standardize TLS identification, which is another powerful weapon against cancer in clinical practice. Moreover, the current TLS identification method can only be carried out using tissue biopsy. Identifying a certain feature from peripheral tissues (such as blood, digestive juice, and even excreta) will reduce patient damage and improve detection efficiency.

Second, TLS needs to be studied from a wider angle and not just as a prognostic marker. If possible, it should be used to monitor the treatment effect during a treatment intervention. Of course, less invasive inspection methods for this process would be important. Furthermore, whether the formation and composition of TLS of PHC caused by different pathogenic factors is different (such as cancer caused by HBV/HCV-related chronic hepatitis versus cancer caused by chemical factors) and whether iTLS and pTLS will transform or promote each other need further study; these will be very significant in understanding TLS evolution and disease causes.

It cannot be ignored that TLS research needs to be translated into PHC immunotherapy, which may solve the problem of the low response rate to ICIs in PHC, thus benefiting patients. As mentioned in the paper, research on both TLS and immunotherapy should be multi-faceted. It may be possible to screen sensitive patients for a certain type of immunotherapy drug or induce its generation and consumption to regulate the efficacy of immunotherapy. Combination therapy is also a potential effective treatment. However, more in-depth mechanism research and verification *via* large-scale *in vivo* and *in vitro* experiments are required.

Finally, the defects in immunodeficient mice used in TLS research are not suitable for investigating the controversial TLS effects in PHC. Further research should focus on how to reconstruct the complex TME of tumor tissue in the human body while considering the interaction of various factors in the body’s immunity.

## Author Contributions

WJ and TZ have made similar contributions to the design and conception of review. QY has carefully reviewed the first draft of the article. JL, YN, WShi, and XL contributed to the discussion. WSong supervised all aspects of the literature review design and manuscript writing. All authors contributed to the article and approved the submitted version.

## Funding

This study was supported by the National Natural Science Foundation of China (81672716).

## Conflict of Interest

The authors declare that the research was conducted in the absence of any commercial or financial relationships that could be construed as a potential conflict of interest.

## Publisher’s Note

All claims expressed in this article are solely those of the authors and do not necessarily represent those of their affiliated organizations, or those of the publisher, the editors and the reviewers. Any product that may be evaluated in this article, or claim that may be made by its manufacturer, is not guaranteed or endorsed by the publisher.
